# Implication of IL-18 in chronic inflammation of severe refractory asthma

**DOI:** 10.1186/2045-7022-5-S2-O10

**Published:** 2015-03-23

**Authors:** Nikoletta Rovina, Efrosini Dima, Petros Bakakos, Eleni Tseliou, Konstantina Kontogianni, Spiridon Papiris, Nikolaos Koulouris, Stylianos Loukides

**Affiliations:** 1 1st Department of Pulmonary Medicine, Sotiriah Hospital, Athens Medical School, Athen, Greece; 2 Sotiria Hospital, Athens Medical School, 1st Department of Pulmonary Medicine, Athens, Greece; 3 Sotiria Hospital, Athens Medical, 1st Department of Pulmonary Medicine, Athens, Greece; 4 Sotiria Hospital, Athens Medical School, 2nd Department of Pulmonary Medicine, Athens, Greece; 5 Sotiria Hospital, Athens Medical School, 2nd Department of Pulmonary Medicine, Athens, Greece

## Background

Bronchial asthma is traditionally viewed as an eosinophilic airway inflammatory disorder with the involvement of Th2 cytokines. In refractory asthma, where chronic inflammation and airway remodeling are key pathological features, it is suggested that the dysregulation of the Th1/Th2 cytokines production modifies airway inflammation and airway hyperrespnsiveness (AHR).

Interleukin (IL)-18, a pleiotropic proinflammatory cytokine, contributes to the pathophysiology of asthma by modulating airway inflammation. Aim of this study was to investigate IL-18 levels in refractory asthma and its relation to eosinophilic airway inflammation and airway remodelling.

## Method

IL-18 levels were measured in sputum supernatants obtained from patients with mild asthma (21 smokers and 24 non smokers), and patients with refractory asthma (n=18). Eosinophilic airway inflammation was assessed by measuring ECP, eosinophil counts in sputum and AHR to methacholine. Airway remodelling was assessed by measuring IL-13, VEGF and transforming growth factor (TGF)-b1 in induced sputum.

## Results

Patients with refractory asthma had significantly lower IL-18 levels in sputum compared to smoking and non smoking asthmatics (p<0.001). IL-18 levels were correlated to the macrophages percentage (r=0.635, p=0.026) and inversely correlated to neutrophils percentage in sputum (r=-0.715, p=0.009). Furthermore, IL-18 levels in sputum correlated to FEV_1_ (% pred) (r=0.760, p=0.004), to FVC (% pred) (r=0.620, p=0.032), and to PD20Mch (r=0.733, p=0.007). No correlations were found between IL-18, ECP, TGF-1, VEGF levels, and eosinophil counts in the sputum of refractory asthma.

## Conclusion

These findings suggest that in refractory asthma IL-18 is possibly involved in chronic airway inflammation and airway remodelling through an eosinophil independent pathway. The decreased levels of IL-18 in refractory asthma might be the result of impaired inflammasome activation in this asthma phenotype, justifying the susceptibility of these patients for infectious exacerbations.

